# Supplementary material for: Anti-Proliferative, Anti-Angiogenic and Safety Profiles of Novel HDAC Inhibitors for the Treatment of Metastatic Castration-Resistant Prostate Cancer
Source: Pharmaceuticals (Basel). 2021 Oct 4;14(10):1020. doi: 10.3390/ph14101020 (PMC8540814; doi:10.3390/ph14101020)
Supplement: Supplementary file 1 [file pharmaceuticals-14-01020-s001.zip › pharmaceuticals-1359820-supplementary.pdf]

pharmaceuticals

MDPI

Supplementary file

Anti-Angiogenic and Safety Profiles of Novel HDAC Inhibitors

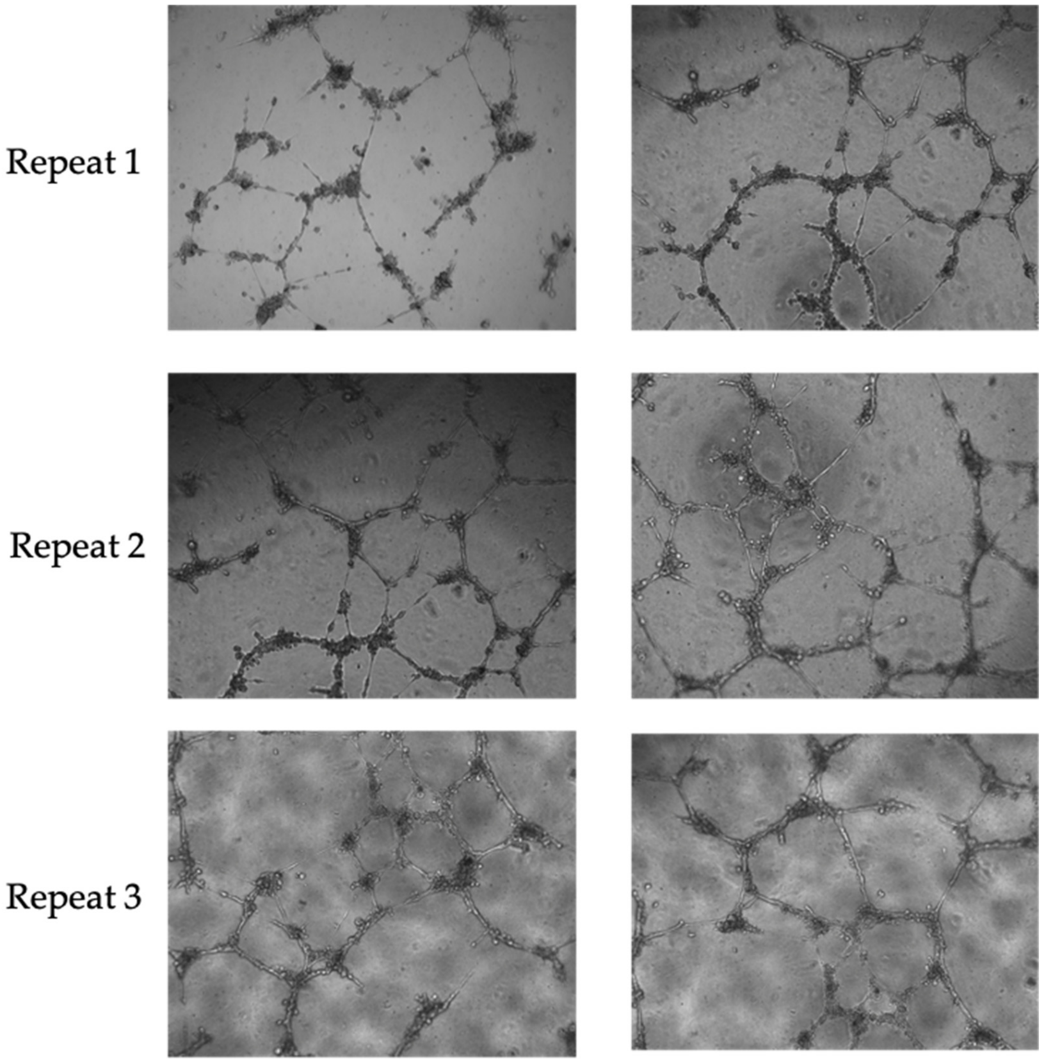

Figure S1. Images captured during the HUVEC tube formation assays for the treatment of the control. These images were analysed using the angiogenesis analyser plugin to measure the total segment length and total number of meshes in ImageJ.

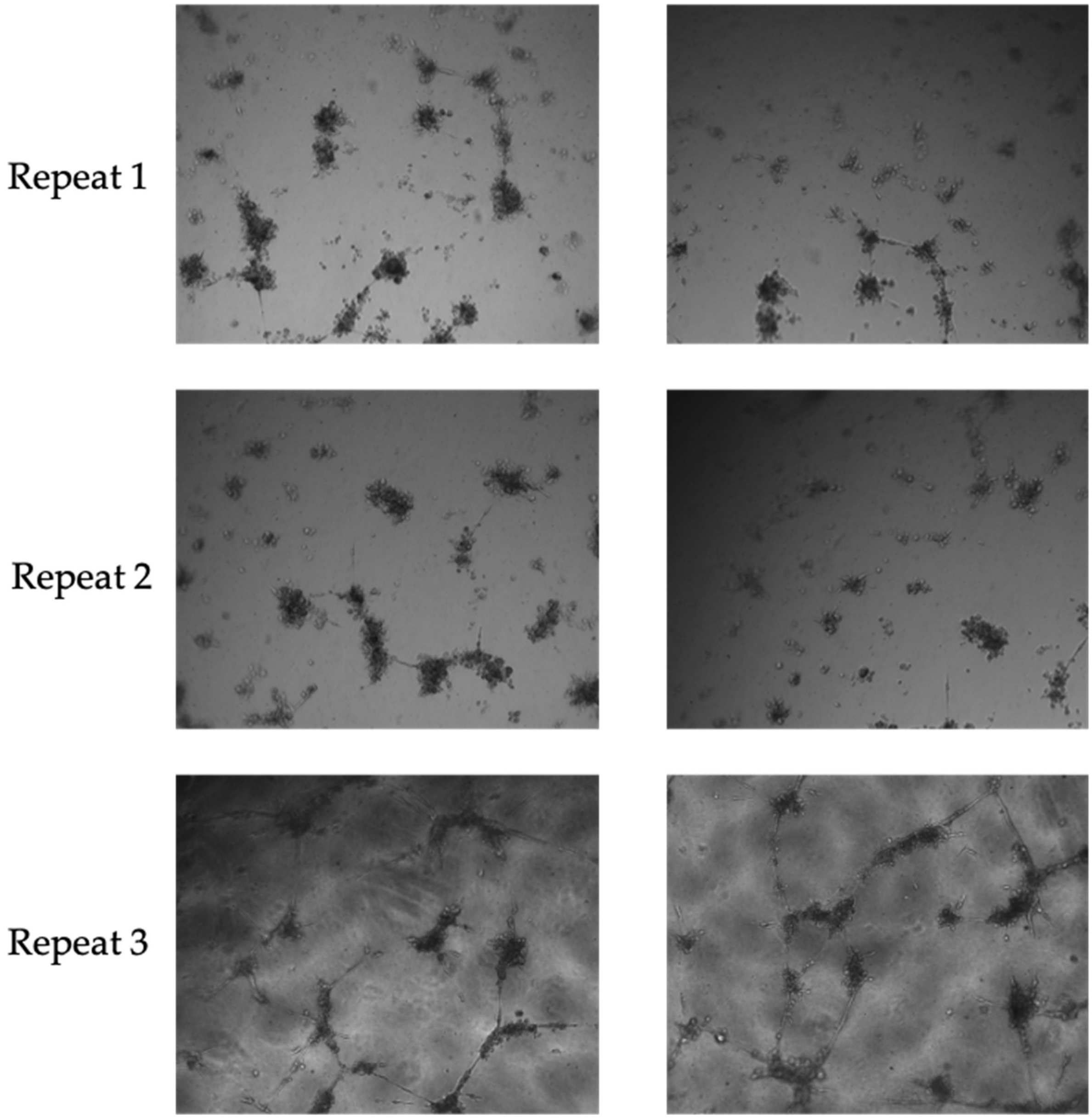

Figure S2. Images captured during the HUVEC tube formation assays after treatment with SAHA at 4 μM. These images were analysed using the angiogenesis analyser plugin to measure the total segment length and total number of meshes in ImageJ.

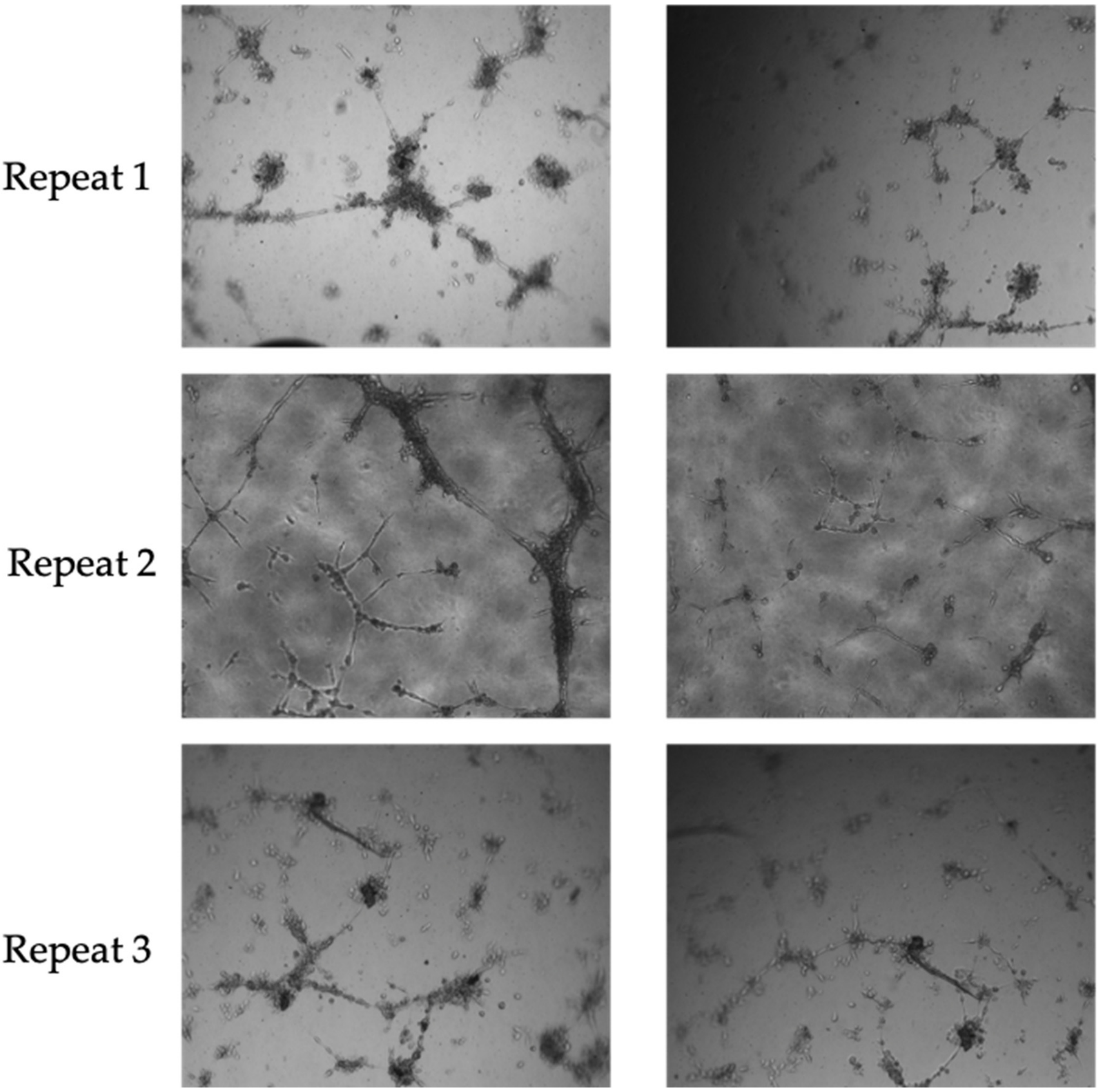

Figure S3. Images captured during the HUVEC tube formation assays after treatment with Jazz90 at 4 μM. These images were analysed using the angiogenesis analyser plugin to measure the total segment length and total number of meshes in ImageJ.

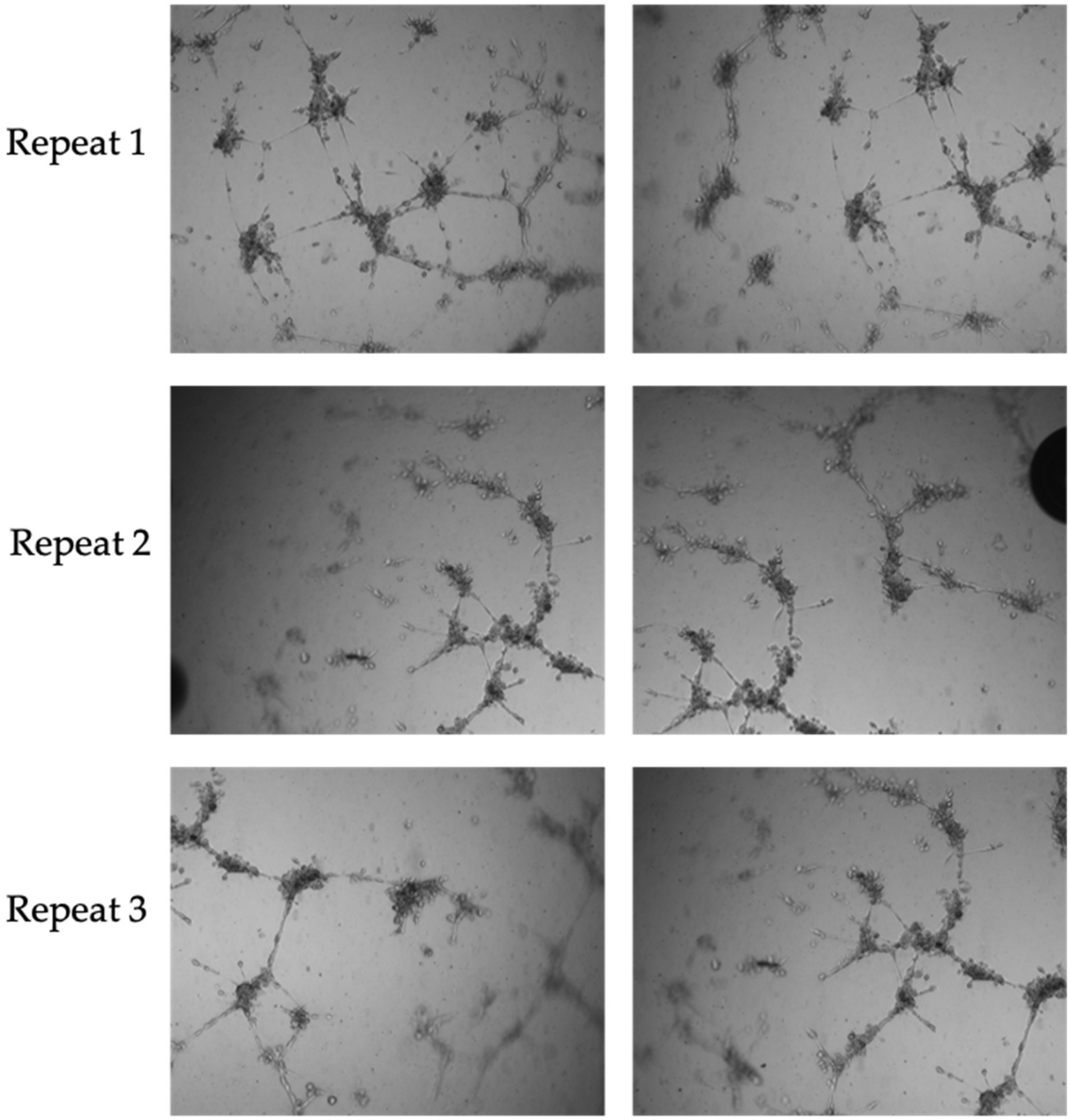

Figure S4. Images captured during the HUVEC tube formation assays after treatment with Jazz167 at 4 µM. These images were analysed using the angiogenesis analyser plugin to measure the total segment length and total number of meshes in ImageJ.

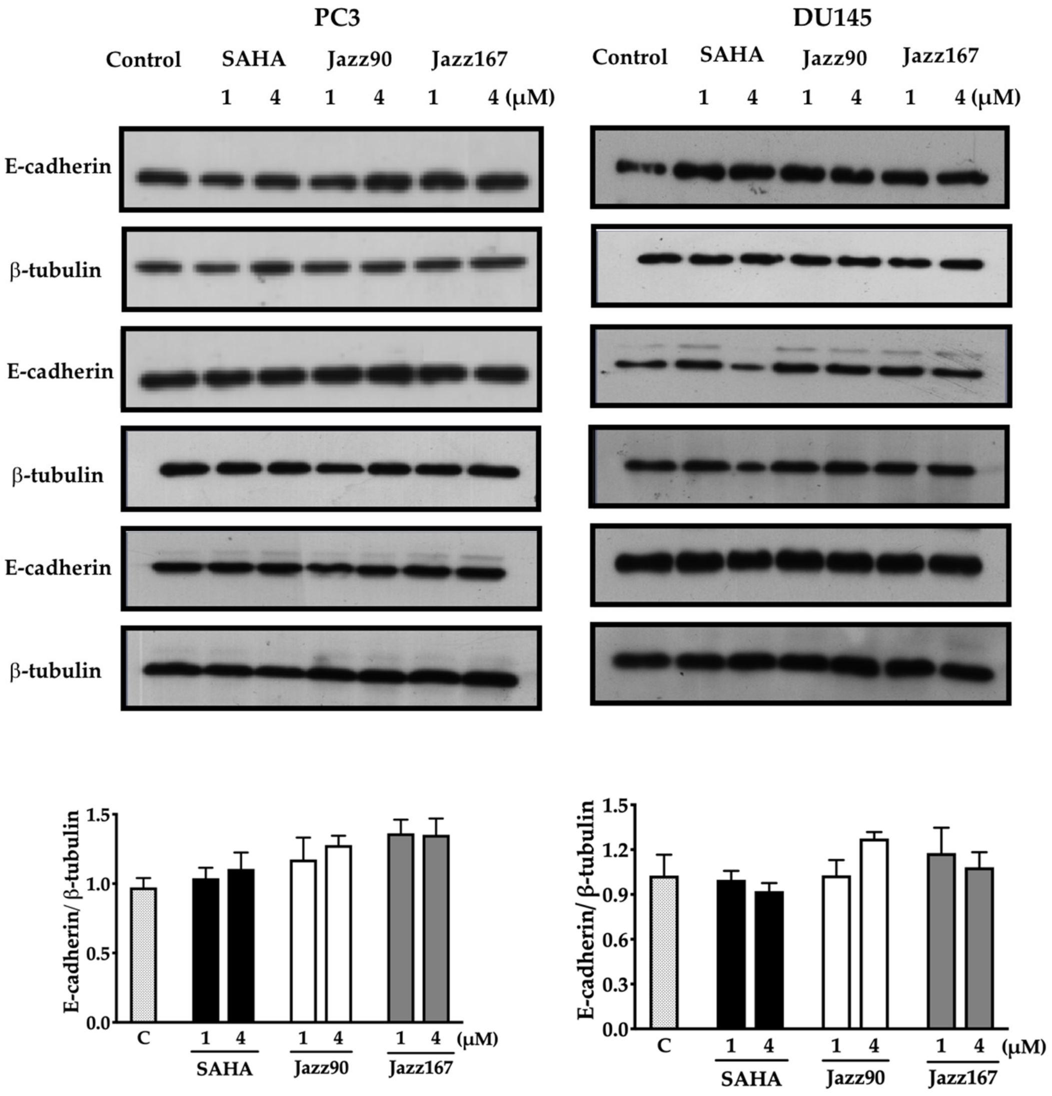

Figure S5. Expression profile of epithelial protein, E-cadherin, in PC3 and DU145 cells. No significant differences were found between the expression of Bcl-2 in drug treated cells compared to vehicle control. Mean (+/-) S.E.M was calculated from 3 independent experiments. Two way ANOVA with Bonferroni's posthoc test was used to measure statistical differences. None were significantly different.

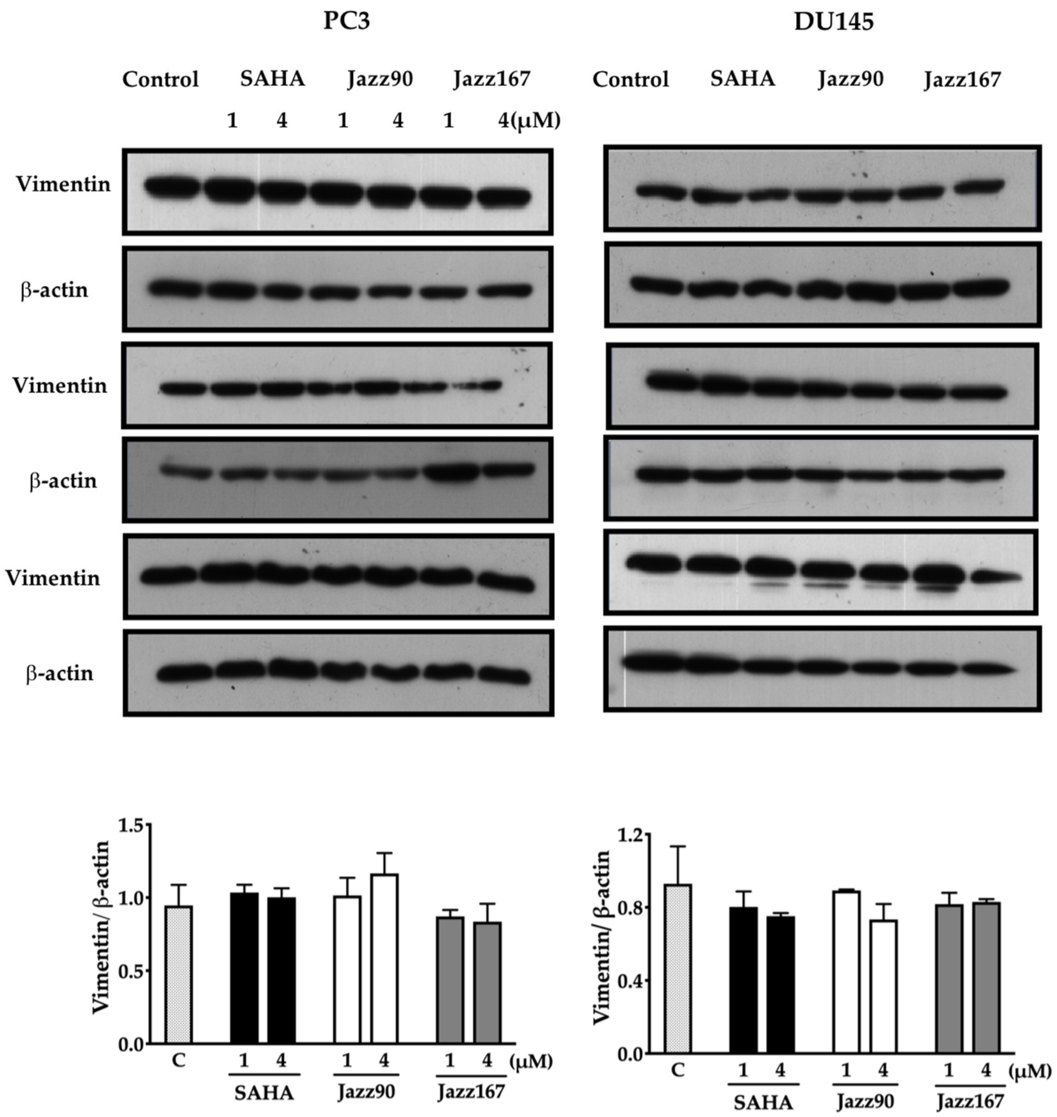

Figure S6. Expression profile of mesenchymal protein, vimentin, in PC3 and DU145 cells. No significant differences were found between the expression of Bcl-2 in drug treated cells compared to vehicle control. Mean (+/-) S.E.M was calculated from 3 independent experiments. Two way ANOVA with Bonferroni's posthoc test was used to measure statistical differences. None were significantly different.

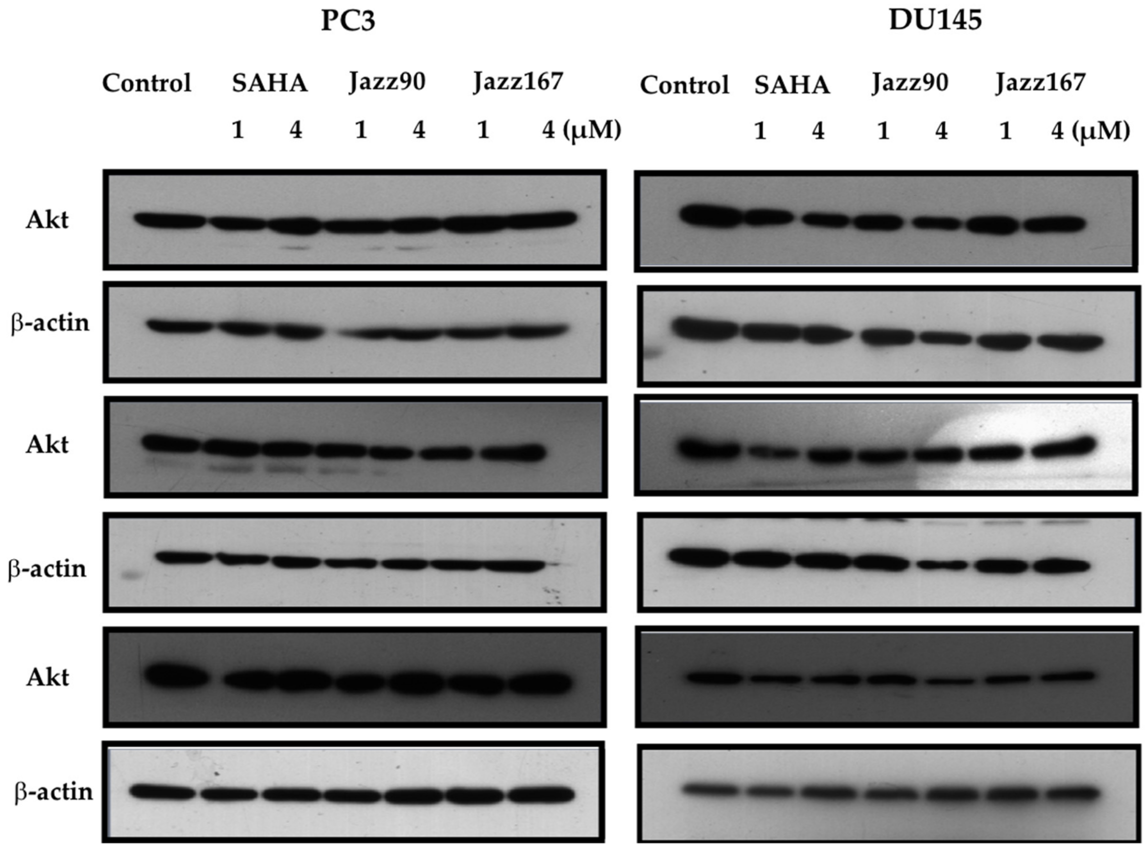

Figure S7. Blots indicating the levels of Akt relative to β-actin in PC3 and DU145 cells. These blots were assessed via densitometry.

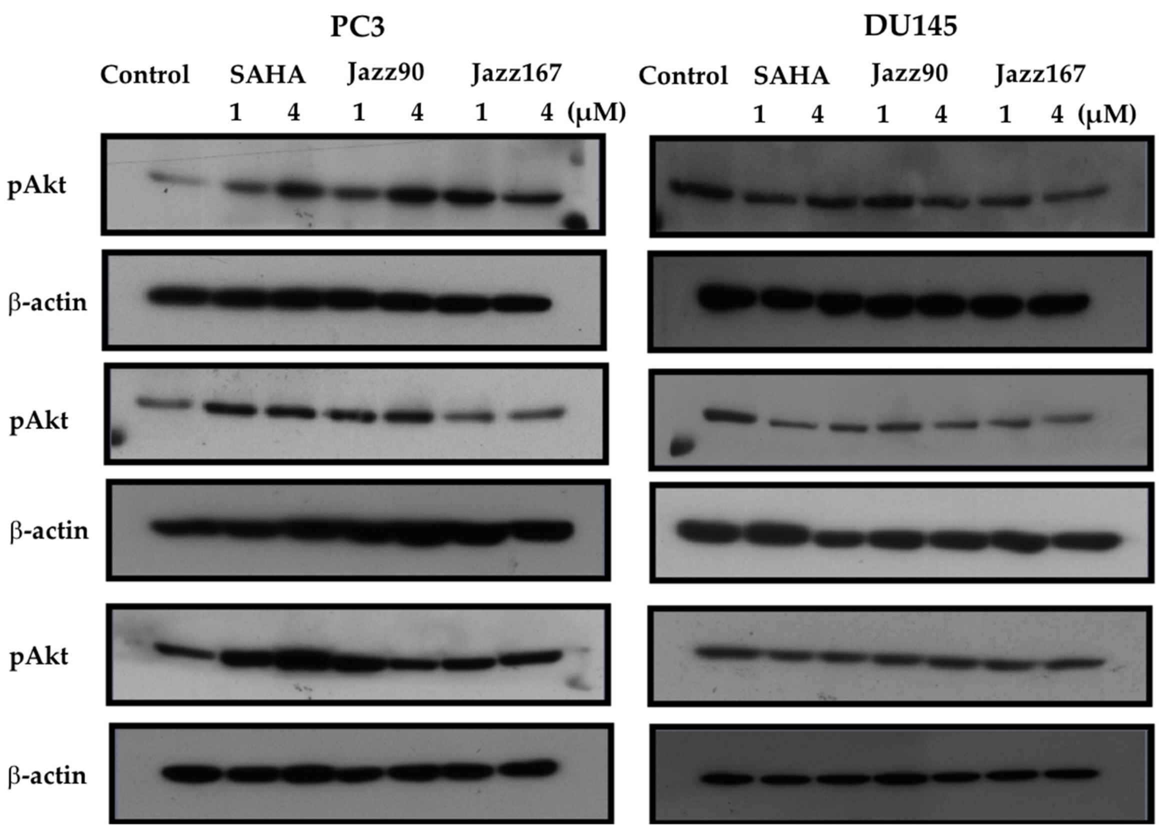

Figure S8. Blots indicating the levels of phosphorylated Akt (pAkt) relative to β-actin in PC3 and DU145 cells. These blots were assessed via densitometry.

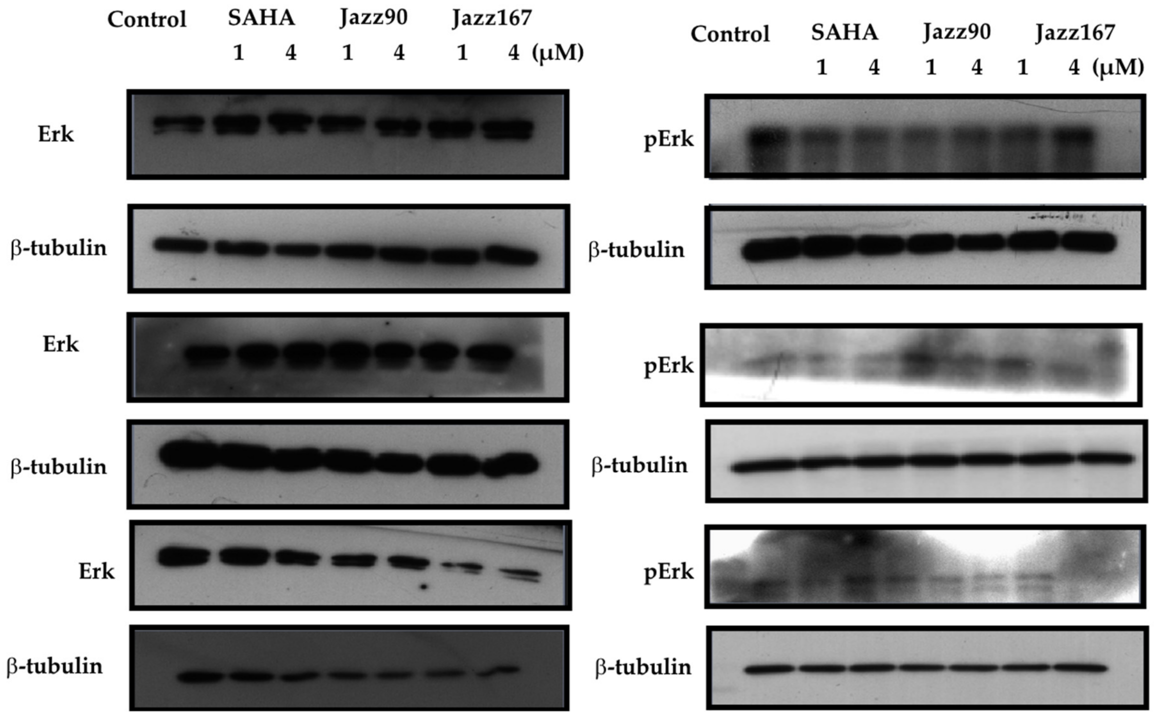

Figure S9. Blots indicating the levels of Erk and phosphorylated Erk (pERK)in PC3 cells. These blots were assessed via densitometry.
